# Recruitment of bone marrow-derived cells to periodontal tissue defects

**DOI:** 10.3389/fcell.2014.00019

**Published:** 2014-05-21

**Authors:** Yasuyuki Kimura, Motohiro Komaki, Kengo Iwasaki, Masataka Sata, Yuichi Izumi, Ikuo Morita

**Affiliations:** ^1^Department of Periodontology, Graduate School of Medical and Dental Science, Tokyo Medical and Dental UniversityTokyo, Japan; ^2^Department of Nanomedicine (DNP), Graduate School of Medical and Dental Science, Tokyo Medical and Dental UniversityTokyo, Japan; ^3^Department of Cardiovascular Medicine, Institute of Health Biosciences, The University of Tokushima Graduate SchoolTokushima, Japan; ^4^Global Center of Excellence Program, International Research Center for Molecular Science in Tooth and Bone Diseases, Tokyo Medical and Dental UniversityTokyo, Japan; ^5^Department of Cellular Physiological Chemistry, Graduate School of Medical and Dental Science, Tokyo Medical and Dental UniversityTokyo, Japan

**Keywords:** bone marrow chimeric mice, periodontal defects, mesenchymal stem cells, recruitment, SDF-1

## Abstract

Bone marrow-derived cells (BMCs) are considered to be a major source of mesenchymal stem cells (MSCs) in adults and are known to be effective in periodontal tissue regeneration. However, whether endogenous BMCs are involved in periodontal tissue repair process is uncertain. We therefore created periodontal tissue defects in the buccal alveolar bone of mandibular first molars in bone marrow chimeric mice, and immunohistochemically examined the expression of stromal cell derived factor-1 (SDF-1) and the mobilization of BMCs. We found that SDF-1 expression was increased around the defects at as early as 1 week after injury and that BMCs were mobilized to the defects, while GFP+/CD45+ were rarely observed. Fluorescence-activated cell sorting (FACS) analysis demonstrated that the number of platelet-derived growth factor receptor (pdgfr) α+/Sca-1+ (PαS) cells in the bone marrow decreased after injury. Taken together, these results suggest that BMCs are mobilized to the periodontal tissue defects. Recruitment of BMCs, including a subset of MSCs could be a new target of periodontal treatment.

## Introduction

Periodontal disease is a bacterially induced chronic inflammatory disease that destroys the tooth-supporting tissue and is one of the main causes of tooth loss. The inflammation and breakdown of tissue can be prevented by conventional periodontal treatment such as scaling and root plaining (tissue debridement). However, the conventional treatment does not regain the tissue that has been lost during the disease process. Recently MSC-like cells have been discovered in periodontal ligament (PDLSCs) (Seo et al., 2004) and extensive studies have been carried out to investigate the potential use of PDLSCs as a therapeutic agent for periodontal regeneration.

Mesenchymal stem cells (MSCs) show multi-differentiation capability and self-renewability *in vitro*. Bone marrow is considered as the one of main source of MSCs. Bone marrow MSCs have attracted attention as donor cells for regenerative therapy, and the efficacy of bone marrow MSCs has been also shown by experiments in the periodontal tissue regeneration. For example, it has been reported that canine periodontal defects can be regenerated from bone marrow MSCs mixed with atelocollagen (Kawaguchi et al., 2004) and that transplanted bone marrow MSCs were observed in regenerated periodontal tissue (Hasegawa et al., 2006). Yang et al. demonstrated that engraftment of bone marrow-derived MSCs with gelatin beads successfully regenerated periodontal tissue in rats (Yang et al., 2010).

Endogenous bone marrow-derived cells (BMCs) including MSCs have been reported to promote repair of the remote tissue by mobilizing into peripheral blood by injury signals, and homing to injured tissues (Mansilla et al., 2006; Alm et al., 2010). Recently, SDF-1 has been reported to involve in the recruitment and engraftment of stem cells in wound sites (Yin et al., 2010). C-X-C chemokine receptor type 4 (CXCR4) is a unique receptor of SDF-1, and is known to express in various cell types, including stem cells (Honczarenko et al., 2006). However, whether the SDF-1/CXCR4 axis is involved in recruitment of BMCs to periodontal wound is not clarified. Particularly, the contribution of endogenous bone marrow MSCs during periodontal tissue repair is not fully understood due to lack of appropriate detection system of MSCs *in vivo*.

A lack of the unique marker is a major obstacle for detection of MSCs *in vivo*. The characteristics of MSCs were confirmed by combination expression of cell surface markers such as CD44, CD73, CD90, and CD105 in a single cell. Besides it must be shown that there is not expression of hematopoietic stem cell marker, CD34 and hematopoietic progeny markers such as CD11b, and CD45. Recently, Morikawa et al. reported the method to isolate MSCs from murine bone marrow without cell culture by cell sorting of pdgfrα (+)/Sca-1 (+) cells (Morikawa et al., 2009).

In this study, we created periodontal defects in the buccal alveolar bone of mandibular first molars in bone marrow chimeric mice and investigated the expression of SDF-1 and the recruitment of bone marrow MSCs during periodontal tissue repair process.

## Materials and methods

### Preparation of bone marrow chimeric mice

BMCs were isolated from femurs and tibias of GFP transgenic mice as reported previously (Okabe et al., 1997; Sata et al., 2002; Fukuda et al., 2009). In brief, BMCs were hemolyzed with ACK lysing buffer (Lonza, Basel, Switzerland). C57/BL/6 mice (age, 8 weeks; male) were lethally irradiated 9.5 Gy (MBR-1520RB; Hitachi, Tokyo, Japan). Two days after irradiation, unfractionated BMCs (1 × 10^6^ cells/0.3 ml D-PBS) from GFP transgenic mice were intravenously injected into irradiated mice by tail vein puncture. Eight weeks after the transplantation, peripheral bloods were collected from retro-orbital plexus. Replacement ratio of bone marrow was confirmed by fluorescence-activated cell sorting (FACS) aria (BD, Franklin Lakes, NJ, USA). Chimeric mice with a bone marrow substitution rate of over 83% were used in this experiment. All procedures involving the experimental animals were performed in accordance with protocols approved by the local institutional guidelines for animal care of The University of Tokyo and Tokyo Medical and Dental University (0120218A) and complied with the *Guide for the Care and Use of Laboratory Animals* (NIH guidelines 32).

### Experimental periodontal tissue injury

Under general anesthesia with sodium pentobarbital (40–50 mg/kg, IP), we produced 2.0 × 1.5 mm periodontal tissue defects in the buccal alveolar bone of mandibular first molars in bone marrow chimeric mice, by removing alveolar bone, periodontal ligament and cementum using a round bar with water cooling under a stereoscopic microscope.

### Immunohistochemical staining

Before or at 1, 2, 4, 5, and 10 weeks after injury, tissue was fixed with 4% paraformaldehyde, followed by decalcification in 10% ethylenediaminetetraacetic acid (EDTA) solution at 4°C. Tissue sections (5 μ) were immunostained for GFP and SDF-1. Briefly, after deparaffinization, sections were washed with PBS, and were treated with 1% hydrogen peroxide (Wako, Osaka, Japan) in methanol. After washing with PBS, sections were blocked with blocking solution (0.5% goat serum in PBS) at room temperature for 30 min.

Before anti mouse SDF-1 antibody treatment, sections were treated with a M.O.M. Immunodetection Kit (Vector Laboratories Inc., Burlingame, CA, USA). Sections were then treated with either anti-mouse GFP antibody (1: 500 dilution, #47894A; Molecular Probes, Eugene, OR) or anti mouse SDF-1 antibody (1: 1000 dilution, #79014; R&D systems, Minneapolis, MN, USA) or anti-mouse CD45 antibody (1: 200 dilution #550539; BD) for 2 h. After washing with PBS three times, sections were treated with secondary antibodies, either anti-rabbit conjugated with biotin (Dako Japan, Kyoto, Japan) or anti-mouse conjugated with biotin (Dako Japan) or anti-rat Cy3 (1: 200 diluted, #56021; Jackson, West Grove, PA, USA) at room temperature for an hour. After washing with PBS three times, sections were treated with ABC-AP mix (Vector Laboratories Inc.) at room temperature for an hour. Detection was performed using Vector Red at room temperature for 10–20 min. Histological examination was performed under a fluorescence microscope (BZ-8000; Keyence, Osaka, Japan)

### Fluorescence-activated cell sorter (FACS) analysis for identification of bone marrow MSCs

In the experimental group, BMCs were obtained from murine femurs and tibias 1 week after preparation of periodontal defects. Untreated mice were used as controls. To evaluate the ratio of bone marrow MSCs for all collected cell counts, we conducted at least three experiments using two mice in the experimental and control groups. BMCs were obtained as described previously (Morikawa et al., 2009). Briefly, femurs and tibias were aseptically removed from two mice and bones were crushed with an ice-cold pestle and mortar. Bone chips, including marrow were rinsed with HBSS+ and were digested using collagenase (#032-22364; Wako) for an hour at 37°C. Collected BMCs were hemolyzed and FcR was blocked with anti-mouse CD16/32 (#553142; BD) on ice for 5 min. BMCs (2–5 × 10^5^) were multi-stained with CD45.2-APC-eFlour780 (#47-0454; eBioscience, San Diego, CA, USA), TER119-PECy7 (#25-5921; eBioscience), pdgfrα-PE (#12-1401-81; eBioscience), Sca-1-APC (#17-5981-81; eBioscience) (1: 100 dilution, 30 min, on ice) and 7AAD (#559925; BD) (1: 100 dilution, 10 min, on ice). The ratio of MSCs [CD45.2 (−), TER119 (−), 7AAD (−), pdgfrα (+), Sca-1 (+)] in BMCs was evaluated by FACS analysis.

## Results

### Localization of GFP-positive cells in periodontal tissue

**Figure 1:**
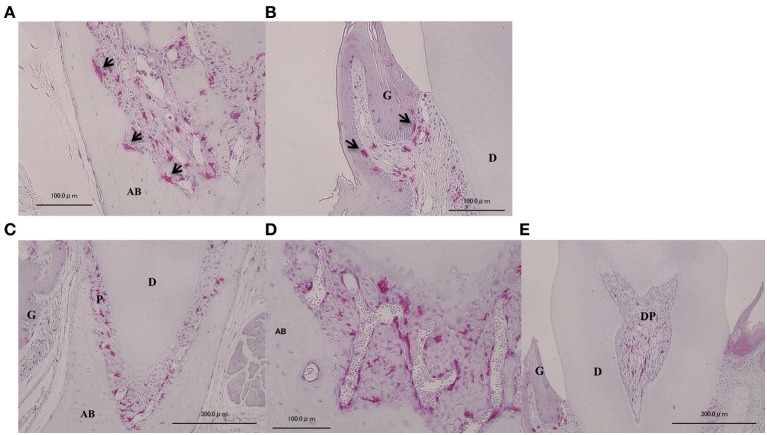
**Localization of GFP-positive cells in intact periodontal tissue**. Visualization of BMCs *in vivo* on periodontal tissue. Arrows indicate osteoclast-like multinucleated giant cells **(A)** and macrophage-like cells in gingival epithelium **(B)** were GFP-positive, implying that GFP-positive images were confirmed to match BMCs. GFP-positive cells were also observed at the periodontal ligament **(C)**, blood vessels **(D)**, and dental pulp **(E)**. A, multinucleated giant cells in resorption lacunae of alveolar bone; B, gingival epithelium; C, periodontal ligament; D, blood vessels in periodontal ligament; E, dental pulp. G, gingiva; AB, alveolar bone; P, periodontal ligament; DP, dental pulp; D, dentin.

In order to investigate the localization of BMCs, we prepared bone marrow chimeric mice and examined GFP-positive cells in the periodontium. Both osteoclasts-like multinucleated giant cell in resorption pits of alveolar bone and macrophages in gingival epithelium, which are known to be derived from bone marrow, were positive for GFP (Figures 1A,B). In control mice, GFP-positive cells were observed in both periodontal ligament and dental pulp (Figures 1C–E). GFP-positive cells in periodontal ligament were mainly observed around blood vessels (Figure 1D). GFP-positive cells were rarely observed in alveolar bone or dentin.

### Time-course observations of BMCs around periodontal tissue defects

**Figure 2:**
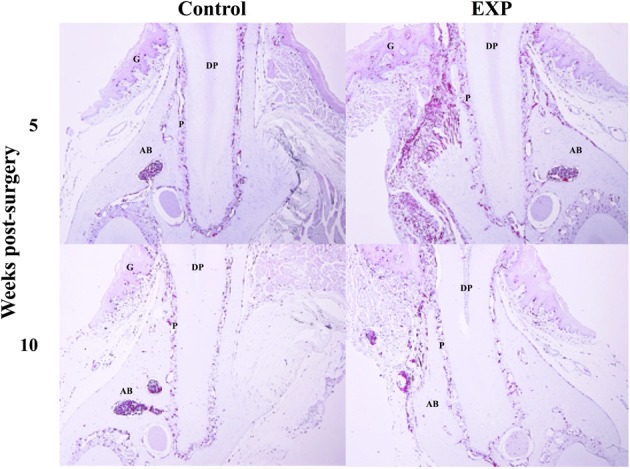
**Time-course observations of BMCs around periodontal tissue defects**. Experimental periodontal tissue defects were introduced at the buccal surface of mandibular first molar roots in bone marrow chimeric mice. Five weeks after surgery, the number of GFP-positive cells was significantly elevated around the defects. Ten weeks after surgery, GFP-positive cells around the periodontal tissue defects decreased to control levels. G, gingiva; AB, alveolar bone; P, periodontal ligament; DP, dental pulp; D, dentin.

**Figure 3:**
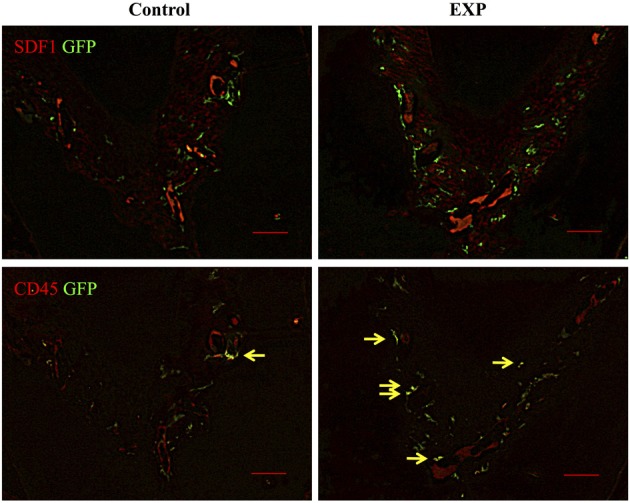
**Double staining of GFP and SDF-1/CD45 in periodontal ligaments**. In control sites, blood vessel-like structures showed intense staining of SDF-1. GFP-positive cells were observed around SDF-1-positive cells. In experimental sites, SDF-1 expression was ubiquitously detected in periodontal ligament 5 weeks after surgery. The number of GFP-positive cells in experimental tissues was higher than in controls. In experimental tissue, the number of GFP(+)/CD45(+) cells (yellow arrows) was elevated, accounting for 13% of GFP-positive cells (*n* = 4).

Experimental periodontal tissue defects were created in bone marrow chimeric mice. Five weeks after injury, GFP-positive cells were observed in the periodontal ligament in both control and experimental groups. In the experimental group, the number of GFP-positive cells increased significantly around the periodontal tissue defects (Figure 2). Ten weeks after injury, GFP-positive cells around the periodontal tissue defects decreased to control levels. Meanwhile, no changes were seen during the course of experiment in control tissue. In addition, we performed double staining for SDF-1 and GFP in order to check co-localization of SDF-1 and GFP. Five weeks after injury, SDF-1 expression was ubiquitously seen in periodontal ligament and the number of BMCs in experimental tissues was higher than in controls (Figure 3). In control tissue, SDF-1 expression was dominantly observed around blood vessels. A double staining for GFP and CD45 showed limited co-localization of GFP (green) and CD45 (red). In experimental tissue the number of GFP(+)/CD45(+) cells peaked at 13% GFP-positive cells.

### Time-course observations of SDF-1 expression around periodontal tissue defects

**Figure 4:**
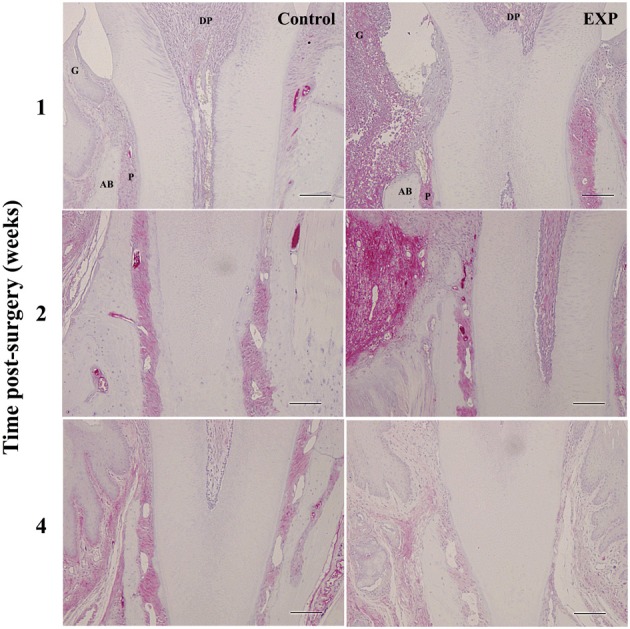
**Time-course observations of SDF-1 expression around periodontal tissue defects**. In experimental sites, SDF-1 expression was slightly higher at 1 week after surgery when compared with controls. SDF-1 expression peaked at 2 weeks after surgery in experimental tissue. SDF-1 expression decreased to control levels by 4 weeks. G, gingiva; AB, alveolar bone; P, periodontal ligament; DP, dental pulp.

At 1, 2, and 4 weeks after injury, SDF-1 expression was immunohistochemically evaluated (Figure 4). One week after injury, some SDF-1 was observed within the blood vessels of periodontal ligaments in control tissue. In experimental tissue, weak and diffuse SDF-1 staining was observed in the defects and periodontal ligament. After 2 weeks, SDF-1 staining increased markedly and was attenuated by 4 weeks after injury.

### Post-operative changes in MSC population in bone marrow

**Figure 5:**
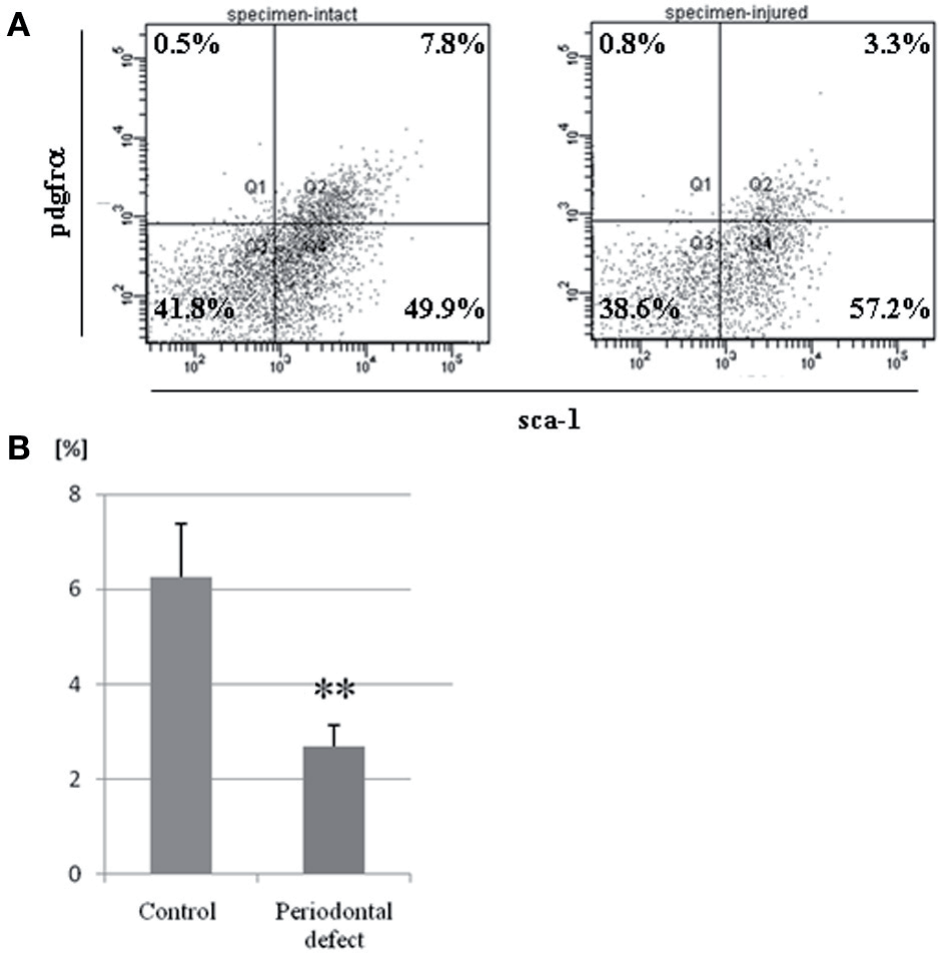
**Postoperative changes in MSC population in bone marrow**. BMCs were collected from murine femurs and tibias in both the control and experimental groups. **(A)** A representative data from FACS analyses. **(B)** FACS analysis showed that the percentage of MSCs in bone marrow of mice with periodontal defects was significantly lower than in controls. (^**^p = 0.01) Independent experiments were repeated at least five times.

One week after injury, BMCs were collected from murine femurs and tibias. Immediately after collecting BMCs, MSC population in bone marrow was evaluated by FACS analysis. FACS analysis confirmed that the percentage of pdgfrα (+)/Sca-1(+) cells in bone marrow of mice with periodontal defects was significantly lower when compared to controls (Figure 5).

## Discussion

Involvement of BMCs in the physiology and pathology of various tissues, including heart, lung, liver, kidney, skeletal muscle, bone, and blood vessels, has been reported. Some of these studies used bone marrow chimeric mice and successfully tracked BMCs. In order to determine roles of endogenous BMCs in periodontal tissue, we created bone marrow chimeric mice. Both osteoclast-like multinucleated giant cells at the alveolar bone and macrophages in gingival connective tissue, known to be of bone marrow origin, were positive for GFP staining (Figures 1D,E), implying BMCs were successfully tracked. However, we should be aware of possibility that recipient bone marrow cells may fuse with donor cells, acquiring such phenotypes in periodontal tissue. GFP-positive cells were observed in both the periodontal ligament and dental pulp (Figures 1A,B). To our knowledge, this is the first time that the presence of endogenous BMCs has been demonstrated in naive murine periodontal ligament, at least by observation of bone marrow chimeric mice. In addition, these cells were mainly observed around blood vessels in periodontal ligament (Figure 1C). McCulloch reported that stem cell-like cells with a slow rate of cell proliferation were located in paravascular sites in murine periodontal ligament (McCulloch, 1985). Chen et al. reported that putative stem cells that showed cross-reactivity with at least one among STRO-1, CD146, and CD44 antibodies, mainly in the paravascular region of human periodontal ligament (Chen et al., 2006). It remains elusive whether BMCs observed in our study were the same as the MSCs/progenitor cells residing in periodontal tissue. It is of interest to clarify the physiological role of BMCs in periodontium and to determine whether BMCs are the origin of MSCs/progenitor cells around blood vessels residing in the periodontal ligament. Further studies are thus necessary.

Next, in order to determine whether BMCs contribute to periodontal wound healing, we created periodontal defects in the bone marrow chimeric mice. Zhou et al. have reported engraftment and differentiation of BMCs to periodontal tissue-forming cells when periodontal defects were treated by regenerative procedure, a grafting of ceramic bovine bone (Zhou et al., 2011). In contrast, Ohta et al. have reported that bone marrow-derived MSCs were not detected in repairing periodontal ligament by a single staining of either STRO-1 or CD44 (Ohta et al., 2008). Our result clearly demonstrated that the number of BMCs around periodontal defects increased as a result of tissue injury in accordance with data reported by Zhou et al. The result that Zhou et al. demonstrated is different from ours in that they investigated the participation of BMCs after periodontal regenerative treatment. In contrast, we investigated whether BMCs are involved in tissue repair without treatment. We observed neither localization of BMCs in alveolar bone 10 weeks post-surgery nor restoration of alveolar bone. It is well known that the tissue debridement abrogates tissue degradation, but does not regain the lost tissue, implying that our model mimics this clinical situation. It is also possible that periodontal tissue healing in irradiated recipient mice may not represent a physiological process that occurs naturally in non-irradiated mice in response to injury. Thus, it remains to be elucidated whether mobilized BMCs actually contribute to periodontal tissue healing.

Kitaori et al. reported that SDF-1 increased during the repair of bone grafts at both the messenger RNA and protein levels, and that anti-SDF-1 antibody inhibited new bone formation (Kitaori et al., 2009). Moreover, Jones et al. demonstrated that migration of MSCs to bone and bone marrow is CXCR4/SDF-1 dependent in a murine osteogenesis imperfecta model and that SDF-1 up-regulates CXCR4 demonstrating chemotaxis *in vitro* and enhancing engraftment *in vivo* (Jones et al., 2012). It has been reported that inflammation and hypoxia play an important role in regulating the SDF-1/CXCR4 axis. First we confirmed that hypoxia around periodontal defects after periodontal injury (supplemental figure 1). We then checked the expression of SDF-1 in periodontium, and found that SDF-1 expression increased around periodontal defects and in periodontal ligament prior to the recruitment of BMCs (Figure 4). It has been reported that SDF-1 levels increase in periodontal disease. (Havens et al., 2008) We confirmed that SDF-1 stimulated the migration of plastic dish-adhered BMCs and that CXCR4-positive BMCs migrated to SDF-1 more promptly than CXCR4-negative BMCs in transwell assay (supplemental figure 2). It has been reported that in BMCs hematopoietic cells, hematopoietic stem cells, MSCs, and endothelial progenitor cells express CXCR4 and that other molecules such as inflammatory factors and cytokines are involved in mobilization of BMCs. (Salem and Thiemermann, 2010) Further experiments are necessary to examine the direct involvement of the SDF-1/CXCR4 axis in recruitment of BMCs *in vivo*. It is also interesting to compare recruitment potential among BMCs.

Adult bone marrow contains several distinct populations of stem cells, such as hematopoietic stem cells, MSCs, endothelial progenitor cells. We observed the number of GFP-positive cells increased in periodontal ligament 5 weeks after injury and about 13% of them are CD45 positive (Figure 3). This result suggested that most of cells detected in periodontal ligament were not hematopoietic cells at least at time point we evaluated (5 weeks after injury). It is necessary to directly determine what types of cells are recruited to periodontal defects.

In FACS analyses, we used CD45 (−), TER119 (−), PDGFRα (+), and Sca-1 (+) cells to detect PαS cells, a subset of MSCs in bone marrow. Based on the results of FACS analyses, we demonstrated for the first time that PαS cells in bone marrow decreased after periodontal injury (Figure 5). However, it was not clarified whether PαS cells contribute to periodontal tissue repair process. Morikawa et al. reported that PaS-derived clones with multi-differentiation potential are also positive for CD105 and CD90 *in vitro* (Morikawa et al., 2009). It is very important to analyze whether PαS cells that migrated to periodontal defects also express CD90 and CD105.

In summary, our data demonstrate that bone marrow cells are mobilized to periodontal defects. Further experiments are needed to determine cells to be mobilized from bone marrow to periodontal defects and regulatory mechanism involved in this process. Recruitment of BMCs, including a subset of MSCs could be a new target of periodontal treatment.

### Conflict of interest statement

The authors declare that the research was conducted in the absence of any commercial or financial relationships that could be construed as a potential conflict of interest.
