# Supplementary material for: Recruitment of bone marrow-derived cells to periodontal tissue defects
Source: Front Cell Dev Biol. 2014 May 21;2:19. doi: 10.3389/fcell.2014.00019 (PMC4207018; doi:10.3389/fcell.2014.00019)
Supplement: Supplementary file 1 [file Presentation1.PDF]

Supplemental figure 1

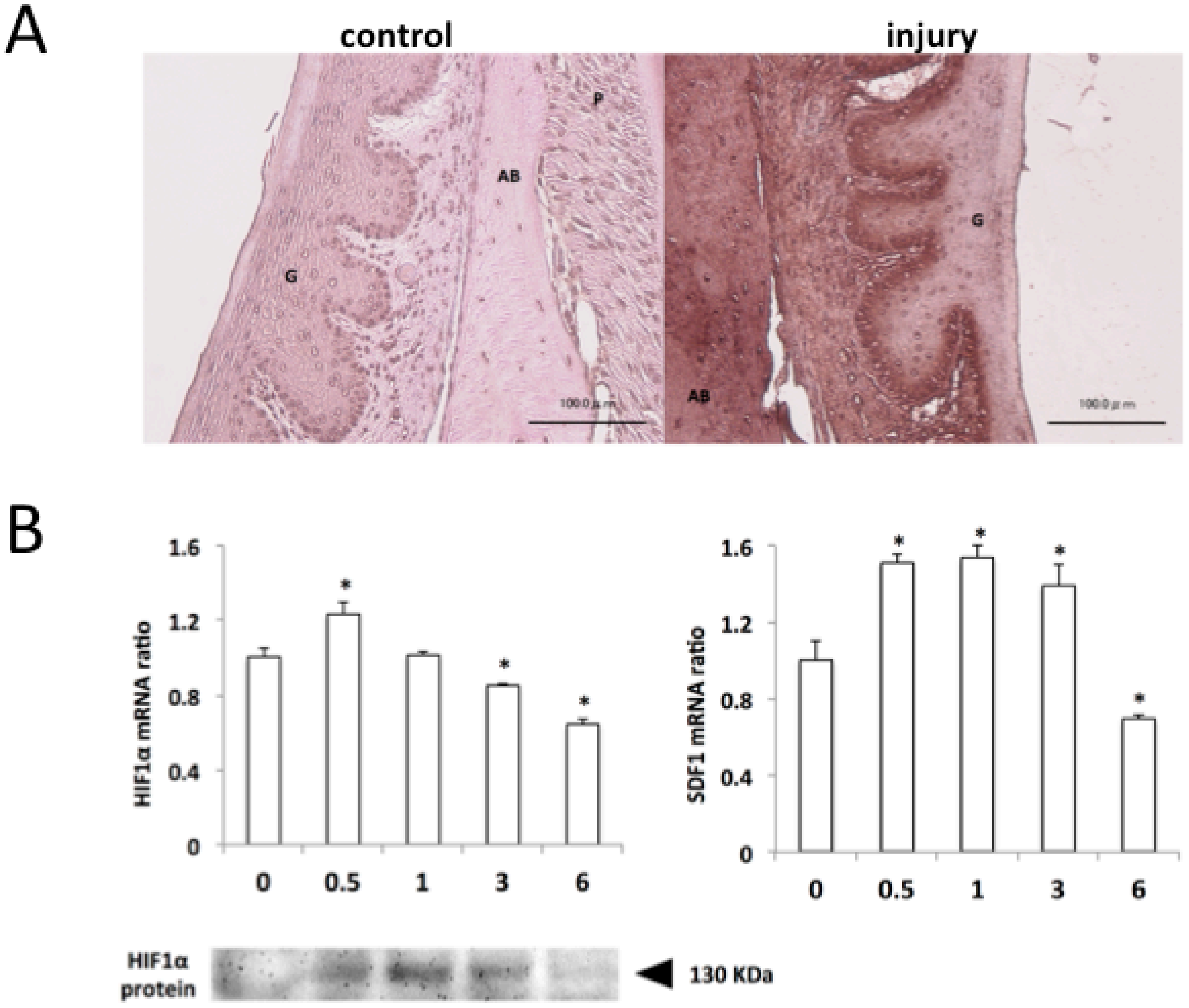

Supplemental figure 1. (A) Hypoxic condition after periodontal injury was evaluated by protein carbonyls. Protein carbonyls are detected immunohistochemistry by using anti-2, 4-dinitrophenylhydrazine antibody (SHIMA laboratories CO., LTD). Periodontal defects were created as described in materials and methods. The tissue section was made 2 days after injury. Periodontal tissue around defects exhibited hypoxia. (B) HIF1α and SDF-1 expression was evaluated. Either total RNA or protein was extracted from mouse endothelial cells after hypoxic stimuli and the expression of HIF1α and SDF1 was evaluated by real time-PCR and western blotting. Note that both HIF1α and SDF-1 were transiently upregulated in endothelial cells. * $p<0.05$.

Supplemental figure 2

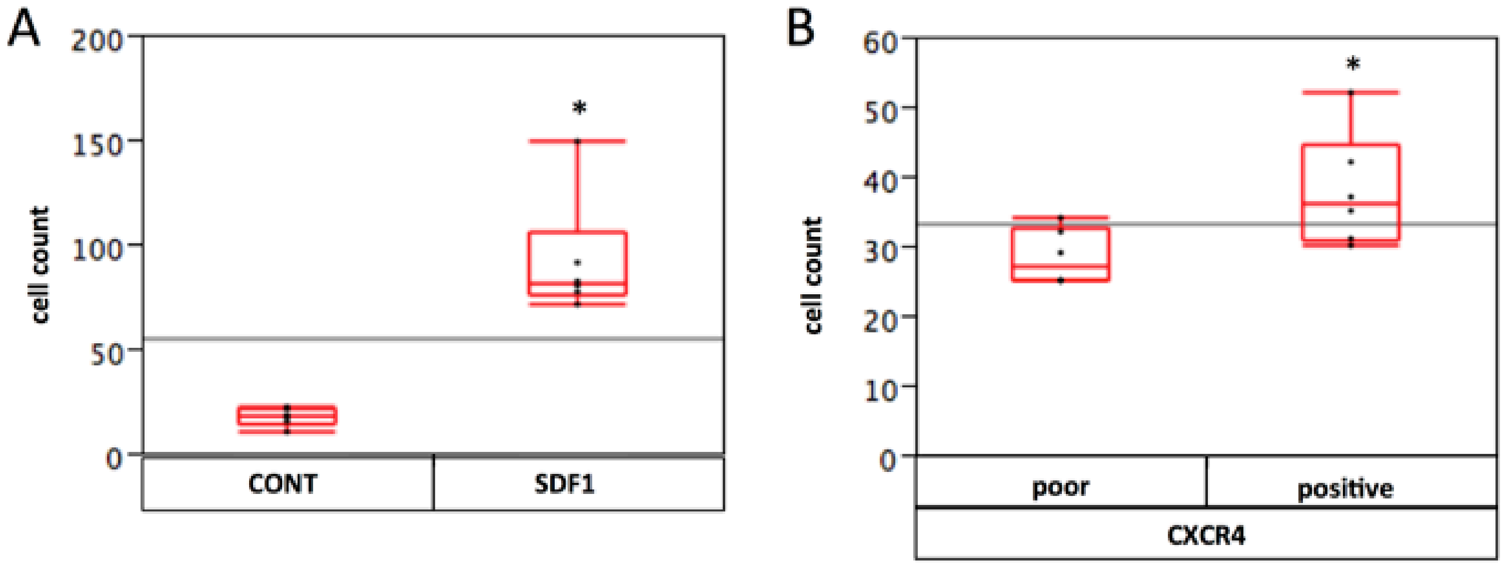

Supplemental figure 2. SDF-1 enhances BMCs migration. BMCs were isolated from intact mice. The chemotactic activity of SDF-1 on BMCs were evaluated by using transwell assay. (A) The number of migrated cells (whole BMCs) 24 hours after SDF-1 addition was counted. (B) BMCs were sorted according to CXCR4 expression by FACS and migration of the cells were compared. Note that BMCs expressing CXCR4 migrated under SDF-1 stimulus more promptly. * $p<0.05$.
